# Neuron myelination is accelerated by Bruton’s tyrosine kinase inhibition via microglial extracellular vesicles

**DOI:** 10.3389/fncel.2026.1864654

**Published:** 2026-10-14

**Authors:** Matteo Tartaglia, Arianna Ioni, Mariassunta De Luca, Laura Vilardo, Igea D’Agnano, Antonella Conte, Cristina Limatola, Myriam Catalano

**Affiliations:** 1 Department of Physiology and Pharmacology, Sapienza University of Rome, Rome, Italy; 2 Department of Human Neurosciences, Sapienza University of Rome, Rome, Italy; 3 Institute of Biomedical Technologies, CNR, Segrate, Italy; 4 IRCCS Neuromed, Pozzilli, Italy; 5 Department of Physiology and Pharmacology, Laboratory Affiliated to Istituto Pasteur Italia Fondazione Cenci Bolognetti, Sapienza University, Rome, Italy

**Keywords:** Bruton's tyrosine kinase, cellular communication, inflammation, microglia, neuroprotection

## Abstract

**Introduction:**

The altered myelin repair observed in demyelinating diseases such as multiple sclerosis (MS) potentially exposes axons to various stressors, with consequent neurodegeneration. Preclinical evidence has demonstrated that Bruton’s tyrosine kinase inhibitors (BTKi), such as ibrutinib, delay MS progression by acting on myeloid cells.

**Methods:**

We investigated the effects of ibrutinib on remyelination using a novel in vitro model of spinal cord neurons derived from postnatal mice.

**Results:**

We demonstrated that (1) spinal cord neuron cells represent a suitable model for investigating myelination following L‑α‑lysophosphatidylcholine (LPC) treatment; (2) ibrutinib induces remyelination and reduces stathmin‑1 expression in spinal cord cultures; and (3) ibrutinib reduces the inflammatory phenotype of microglia‑like BV2 cells, thereby modulating the expression of miR‑223 in extracellular vesicles (EVs).

**Discussion:**

Altogether, these data pave the way for a better understanding of the mechanisms underlying remyelination following ibrutinib treatment and support the hypothesis that EV‑derived microglial cells play a key role.

## Graphical abstract

**Figure:**
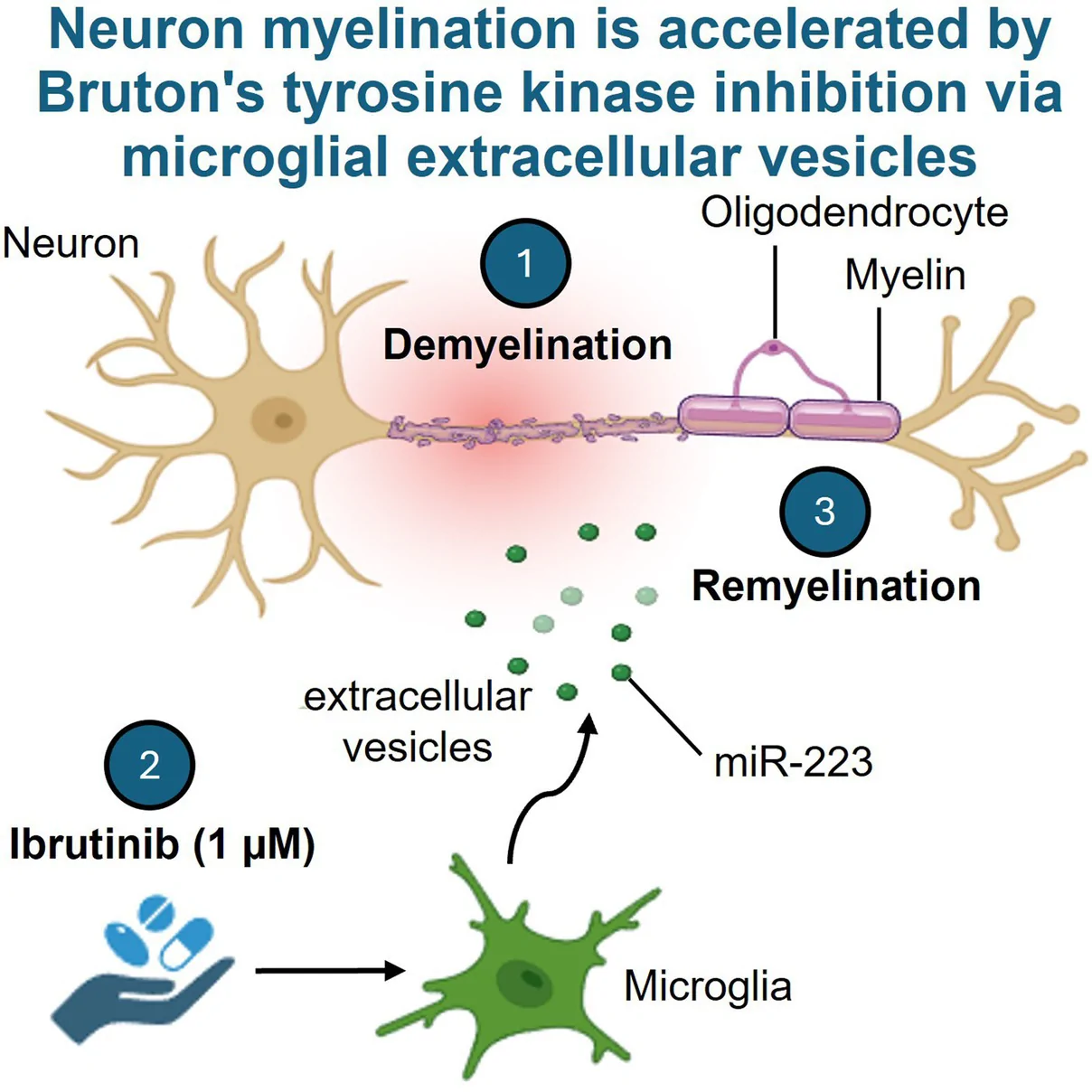

## Introduction

Demyelinating diseases are characterized by damage to the myelin sheath surrounding central nervous system (CNS) axons or to the oligodendrocytes themselves (Love, 2006). This heterogeneous group of disorders can arise from various causes, including infectious agents, hypoxic/ischemic injury, metabolic imbalances, and toxic exposures (Love, 2006).

Despite similarities in neuroimaging findings, demyelinating diseases differ significantly in their distribution, severity, and clinical course (Smith and Smirniotopoulos, 2010).

Among these conditions, autoimmune demyelinating diseases are driven by the immune-mediated destruction of myelin. Both adaptive and innate immune mechanisms contribute to this process. T cells and B cells are central to this damage, while microglia, the resident immune cells of the CNS, play a dual role: they can not only amplify the immune response through antigen presentation but also support tissue repair by clearing debris and releasing neuroprotective factors (Branchi et al., 2014; Cherry et al., 2014). However, in many demyelinating conditions, these reparative mechanisms are insufficient or disrupted (Franklin and ffrench-Constant, 2008).

Emerging evidence suggests that the interaction between microglia and oligodendrocytes plays a significant role in promoting remyelination. A key mediator of this communication is extracellular vesicles (EVs), which carry a variety of signaling molecules, including microRNAs (miRNAs). miRNAs are small non-coding RNAs that regulate gene expression by binding to mRNAs, inhibiting translation or promoting mRNA degradation (Ha and Kim, 2014).

Specific miRNAs, such as miR-155 and miR-223, have been implicated in the regulation of inflammation and repair processes in demyelinating disorders (Mycko and Baranzini, 2020). miR-155 is typically associated with pro-inflammatory responses and is highly expressed in immune cells, such as macrophages and T cells (Xu et al., 2022). While it is known to hinder remyelination by damaging oligodendrocytes, it may also influence immune cell subtypes that support tissue repair, although its net effect on remyelination remains unclear (Wen et al., 2021).

In contrast, miR-223 has been shown to facilitate debris clearance and promote remyelination in models of CNS injury (Ou et al., 2024). It also contributes to an inflammatory milieu in a way that supports regenerative processes (Galloway et al., 2019).

Therapeutic strategies targeting both adaptive and innate immune responses are under investigation. Among these, Bruton’s tyrosine kinase (BTK) inhibitors are gaining attention for their ability to modulate immune cell signaling. BTK is involved in the activation and cytokine production of several immune cell types and is widely expressed in the CNS, particularly in microglia, but is not expressed by oligodendrocytes (Dybowski et al., 2023; Brunner et al., 2005; Yang et al., 2004).

To investigate the potential of BTK inhibition to influence microglia–oligodendrocyte interactions and support remyelination, we employed an *in vitro* model based on primary neural cultures from the brain cortex (BC) and the spinal cord of postnatal mice. This model allowed us to explore both demyelination and remyelination processes. We examined the effects of the BTK inhibitor ibrutinib on murine microglia-like BV2 cells, particularly focusing on their EV release and miRNA content (miR-223 and miR-155). Our findings suggest that ibrutinib may promote the recovery of myelination following L-α-lysophosphatidylcholine (LPC)-induced demyelination in spinal cord cultures. This effect was associated with a reduction in EV release and an increase in miR-223 levels within these vesicles. Additionally, in LPC-treated spinal cord cultures, ibrutinib reduced the expression of *STMN1*, the gene encoding the microtubule-destabilizing protein stathmin-1. Elevated levels of this protein have been observed in oligodendrocytes across several demyelinating conditions, where they may interfere with remyelination (Liu et al., 2005; Jastorff et al., 2009).

These results support the hypothesis that BTK inhibition can modulate microglial function and EV-mediated signaling through mechanisms that may enhance remyelination. Such mechanisms could be relevant across a range of demyelinating diseases, offering a broader therapeutic avenue beyond any single condition.

## Materials and methods

### Primary spinal cord and brain cortex cultures

Male and female C57BL/6 N mouse pups (0–2 days old, P0-2) were used to set up primary neural cell cultures. All methods and related experiments were approved by the Italian Ministry of Health (protocol number: 775/2020) in accordance with the ethical guidelines on the use of animals established by the European Communities Council Directive 2010/63/EU and the Italian Principle of Laboratory Animal Care (D. Lgs 26/2014). All experiments complied with the Animal Research Reporting of *In Vivo* Experiments (ARRIVE) guidelines. Spinal cords were isolated as previously reported (Eldeiry et al., 2017; Sinyuk and Williams, 2020) with minor modifications. From the same animals, brain cortices were also isolated. Tissues were collected in HBSS (Gibco, Thermo Fisher Scientific, Waltham, MA, USA) and cut into small fragments. Brain cortices, after meninges and connective tissue removal, and minced spinal cords were enzymatically treated with trypsin–EDTA (0.1%, Gibco, Thermo Fisher Scientific, Waltham, MA, USA) at 37 °C for 15 min. Immediately, a specific trypsin inhibition solution (in DMEM: 0.05% soybean trypsin inhibitor, Sigma Aldrich, Merck KGaA, Darmstadt, Germany; 0.02% DNase-I, Roche; 0.3% BSA, Sigma Aldrich, Merck KGaA, Darmstadt, Germany) was added; the cells were washed and then centrifuged at 800 x *g* for 5 min. The supernatant was carefully discarded and replaced with plating medium (in DMEM: 50% horse serum, Gibco, Thermo Fisher Scientific, Waltham, MA, USA; 30% DMEM/F-12, Sigma Aldrich, Merck KGaA, Darmstadt, Germany; 20% HBSS-Ca^2+^/Mg^2+^, Gibco, Thermo Fisher Scientific, Waltham, MA, USA), and the tissue was homogenized and then filtered using a 40-μm cell strainer. We obtained 600,000 cells/pup. Approximately 100,000 cells were seeded on poly-L-lysine-coated coverslips (1.32 cm^2^) and incubated at 37 °C, 5% CO_2_. After 2 h, the plating medium was changed to the myelination medium, composed of Neurobasal medium (NBM, Gibco, Thermo Fisher Scientific, Waltham, MA, USA) supplemented with B27 Supplement (1:50, Gibco, Thermo Fisher Scientific, Waltham, MA, USA) and N2 medium in a 1:1 ratio. N2 composition in DMEM-F12 (Sigma Aldrich, Merck KGaA, Darmstadt, Germany) was as follows: transferrin (50 μg/mL, Sigma Aldrich, Merck KGaA, Darmstadt, Germany), sodium selenite (5.2 ng/mL, Sigma Aldrich, Merck KGaA, Darmstadt, Germany), hydrocortisone (18 ng/mL, Sigma Aldrich, Merck KGaA, Darmstadt, Germany), putrescine (16 μg/mL, Sigma Aldrich, Merck KGaA, Darmstadt, Germany), progesterone (6.3 ng/mL, Sigma Aldrich, Merck KGaA, Darmstadt, Germany), biotin (10 ng/mL, PanReac AppliChem, ITW Reagents, Barcelona, Spain), N-acetyl-L-cysteine (5 μg/mL, Sigma Aldrich, Merck KGaA, Darmstadt, Germany), BSA (0.1%, Sigma Aldrich, Merck KGaA, Darmstadt, Germany), and penicillin–streptomycin (1 IU/mL penicillin G, Gibco, Thermo Fisher Scientific, Waltham, MA, USA 1 mg/mL streptomycin, Gibco, Thermo Fisher Scientific, Waltham, MA, USA). From days *in vitro* (DIV)0 to DIV10, myelination medium was supplemented with nerve growth factor (50 ng/mL, Cell Guidance Systems, Cambridge, UK), neurotrophin 3 (10 ng/mL, Cell Guidance Systems, Cambridge, UK), and insulin (10 μg/mL, Selleck Chemicals, Houston, TX, USA), as cells derived from postnatal mice may present slower growth and reduced myelination (Pang et al., 2012). From DIV11, exclusively cortex cultures were supplemented with 3,3′,5-triiodo-L-thyronine (60 ng/mL, Sigma Aldrich, Merck KGaA, Darmstadt, Germany; Pang et al., 2012). To avoid cell overgrowth and consequent detachment, from DIV11, when confluence was reached, both spinal cord and brain cortex cultures were grown in N2: NBM in a 4:1 ratio (Pang et al., 2012).

### Immunofluorescence staining and imaging

The cells were washed with phosphate-buffered saline (PBS), fixed with paraformaldehyde (PFA; 4%, 15 min, RT), permeabilized using Triton X-100 (0.5%, 20 min), and treated with the blocking solution (10% goat serum and 0.1% Triton X-100, 1 h, RT). Primary antibodies (listed in Table 1 at the indicated dilution) were incubated in PBS (o/n, 4 °C), and secondary antibodies (listed in Table 1) were incubated in PBS (1 h, RT). Hoechst 33342 (10 min, RT) was used for nuclei visualization. The coverslips were mounted with a Dako Fluorescence Mounting Medium (Agilent, Santa Clara, CA, USA). Images were acquired using a fluorescence microscope (Nikon, Eclipse Ti-S) or an Abberior STEDYCON confocal/STED system (Abberior Instruments, Göttingen, Germany) mounted on an Olympus IX83 inverted microscope (Olympus Corporation, Tokyo, Japan).

**Table 1:** Primary and secondary antibodies used.

Antibody	Catalog number	Dilution
Primary antibody		
Anti-MBP	Atlas Antibodies HPA019222	1:1000
Anti-Tuj	BioLegend 801,201	1:500
Anti-Olig2	Abnova H0010215M02	1:500
Anti-Iba1	Fujifilm Wako 019–19,741	1:500
Anti-GFAP	Cell Signaling D1F4Q (12389)	1:500
Secondary antibody		
Goat anti-rabbit IgG Alexa Fluor™ 488	Invitrogen A-11034	1:500
Donkey anti-mouse IgG Alexa Fluor™ 488	Invitrogen A-21202	1:500
Goat anti-mouse IgG Alexa Fluor™ 594	Invitrogen A-11032	1:500
Goat anti-rabbit IgG Alexa Fluor™ 594	Invitrogen A-11037	1:500

### Image quantification

Images for each experimental assay were captured using identical camera settings. The images were analyzed in a blinded manner and quantified using MetaMorph 7.6.5.0 software (Molecular Devices, San Jose, CA, USA). For each condition, 10 images were randomly captured from 2–3 coverslips (technical replicates), and their mean value was calculated to generate a single data point, defined as one independent experiment (*n*). The extent of myelination was defined using a direct spatial co-localization criterion. Neurons (identified as Tuj1^+^ cells) were considered myelinated when myelin basic protein (MBP), a protein expressed by mature oligodendroglial cells (Baumann and Pham-Dinh, 2001), enveloped Tuj1^+^ neurites in a single optical plane. The orthogonal projection of the acquired z-stack was used in the analysis to assess the three-dimensional distribution and colocalization of MBP around the neurite. Myelination was expressed as the percentage of myelinated vs. total neuron number.

### Demyelination/remyelination assessment

Primary spinal cord and brain cortex cultures were treated with lysophosphatidylcholine (LPC, 100 μg/mL, dissolved in ethanol, Sigma Aldrich, Merck KGaA, Darmstadt, Germany) at 30-35DIV for 24 h to induce demyelination (Pang et al., 2012; Vereyken et al., 2009). After LPC removal, the cultures were washed and incubated in fresh medium. Ibrutinib (1 μM, dissolved in dimethyl sulfoxide [DMSO], MedChemExpress, Monmouth Junction, NJ, USA) was added 24 h after LPC exposure and subsequently removed after 24 h (Nam et al., 2018). In selected conditions, spinal cord cultures received extracellular vesicles (EVs) isolated from BV2 microglia-like cells (see below). EV administration followed the same temporal scheme used for ibrutinib, with EVs added 24 h after LPC exposure and maintained for 24 h before removal. The amount of EVs applied to cultures corresponded to the EV yield obtained from BV2 donor microglia at a 1:1 donor-to-recipient microglial cell ratio, ensuring that the EV load reflected the microglial fraction physiologically present in the primary cultures. To inhibit endogenous EV release, the cultures were pre-incubated with Calpeptin (Fox et al., 1991; Catalano and O'Driscoll, 2019; 50 μM, dissolved in DMSO; Sigma Aldrich, Merck KGaA, Darmstadt, Germany) for 1 h before LPC exposure. The final concentrations of ethanol or DMSO in the control culture medium were 0.1%. Following drug withdrawal, the cultures were washed again and maintained in ibrutinib-free or EV-free medium for the remaining 7-day recovery period (“LPC rec”). Remyelination was assessed after 7 days (Pang et al., 2012; Vereyken et al., 2009).

### BV2 cells

BV2 murine microglial-like cells were cultured in DMEM supplemented with heat-inactivated 10% FBS, 1 IU/mL penicillin G, 1 mg/mL streptomycin, and 2.5 μg/mL amphotericin B and maintained at 37 °C in a humidified atmosphere with 5% CO2. To induce inflammation, BV2 cells were treated with LPC (100 μg/mL) or LPS (100 ng/mL, Sigma Aldrich, Merck KGaA, Darmstadt, Germany) for 24 h. For EV production for use in spinal cord stimulation, BV2 cells were treated either with LPS (100 ng/mL) or with LPS plus ibrutinib (1 μM) for 24 h. Control cultures received 0.1% DMSO.

### EV extraction and nanoparticle tracking analysis

BV2 supernatant was collected and centrifuged at 1,000 × *g* for 5 min to remove cell debris. The clarified supernatant was then centrifuged at 10,000 × *g* for 30 min at 4 °C, and the supernatant underwent ultracentrifugation at 100,000 × *g* for 1 h at 4 °C. The resulting pellets, containing small EVs, were isolated and resuspended in 0.22 μm-filtered PBS (Thermo Fisher Scientific, Waltham, MA, USA). The dimensions and concentrations of EVs were determined using NanoSight NS300 (Malvern Panalytical, Malvern, UK) technology. The samples were diluted in PBS to a final volume of 1 mL to obtain a range of 20–100 particles per frame. For each independent experiment (*n*), five 60-s movies were recorded. We used the complementary metal-oxide-semiconductor (sCMOS) camera, camera level 14, and detection threshold 4. Videos were analyzed using the integrated NanoSight Software NTA 3.4 Dev Build 3.4.4.

### RT-PCR

Total RNA was extracted from primary cultures and the BV2 cell line with Tri Reagent (Gibco, Thermo Fisher Scientific, Waltham, MA, USA), quantified, and reverse-transcribed using iScript Reverse Transcription Supermix (Bio-Rad Laboratories, Hercules, CA, USA). Real-time polymerase chain reaction (RT-PCR) was carried out in a CFX96 Dx System (Bio-Rad Laboratories, Hercules, CA, USA) using SsoFast Eva Green Supermix (Bio-Rad Laboratories, Hercules, CA, USA). The PCR protocol consisted of 40 cycles of denaturation at 95 °C for 30 s and annealing/extension at 58 °C for 30 s. Fold differences were obtained using the following equation: 2^-ΔCt^, where Ct is the threshold cycle, and ΔCt equals the Ct mean of the sample gene minus the Ct mean of *GAPDH*. Primer sequences: *STMN1*, forward: CGCTTGCGAGAGAAGGACAA, reverse: CGGGACAACTTAGTC AGCCT; *iNOS*, forward: ACATCGACCCGTCCA CAGTAT, reverse: CAGAGGG GTAGGCTTGTCTC; *TNFα*, forward: GTGGAACTGG CAGAAGAG, reverse: CCATAGAACTGATGAGAGG; *GAPDH*, forward: TCGTCCCGTAGACAAAATGG, reverse: TTGAGGTCA ATGAAGGGGTC. Total RNA was extracted from BV2-derived EVs with the Total Exosome RNA & Protein Isolation Kit (Invitrogen, Thermo Fisher Scientific, Waltham, MA, USA) according to the manufacturer’s instructions. RNAs extracted from all samples were quantified and reverse-transcribed using the TaqMan™ MicroRNA Reverse Transcription Kit (Applied Biosystems, Thermo Fisher Scientific, Waltham, MA, USA) according to the manufacturer’s recommendations. Quantitative RT-PCR for miR-223 and miR-155 was performed using TaqMan Universal Master Mix II (Applied Biosystems, Thermo Fisher Scientific, Waltham, MA, USA) on a CFX96 Dx System (Bio-Rad Laboratories, Hercules, CA, USA) and the following two-step cycler profile: 50 °C for 2 min, 95 °C for 10 min, and 40 x (95 °C for 4 s, 60 °C for 1 min). Fold differences were expressed as 2^-ΔCt^, with ΔCt equal to the mean Ct of the sample gene minus the mean Ct of snU6. Primer sequences: hsa-miR-223, UGUCAGUUUGUCAAAUACCCCA; mmu-miR-155, CUCCU ACCUGUUAGCAUUAAC; U6 snRNA, GTGCTCGCTTCGGC AGCACATATAC TAAAATTGGAA CGATACA GAGAAGATTA CATGGCCCCTGCGCAAGGATGACACGCAAATTCGTGA AGCGTTCCATATTTT. For each independent experiment (*n*), the mean of two technical replicates was considered.

### Statistical analysis

For all experiments, data were presented as the mean ± SEM of *n* independent experiments. Dataset normality was assessed using the Kolmogorov–Smirnov test. The comparison of two groups was analyzed using an unpaired two-tailed t-test; comparisons among more than two groups were analyzed using a one-way analysis of variance (ANOVA), followed by Tukey’s multiple comparison tests. For experiments involving two independent variables (e.g., treatment × time), data were analyzed using a two-way ANOVA, followed by Tukey’s multiple comparison test. Non-parametric paired data (EV quantification in BV2 cells) were analyzed using the Wilcoxon matched-pairs rank test. Statistical analyses were performed using Prism (GraphPad Software, San Diego, CA, USA, version 10.4.1). Significance thresholds and exact *p*-values are reported in the corresponding figure captions.

### Image Preparation

All graphs were generated using Prism (GraphPad software, San Diego, CA, USA, version 10.4.1), while the graphical abstract was created with BioRender.com and subsequently refined in Microsoft PowerPoint.

## Results

### Myelination in postnatal neuronal cultures

To validate an *in vitro* model to study demyelination and remyelination using postnatal mice instead of embryos, we set up primary cultures from the spinal cord (SC) and the brain cortex (BC) of P0-P2 mice. Immunofluorescence analysis for cell-specific markers (Olig2 for oligodendrocytes, Tuj1 for neurons, Iba1 for microglia, and GFAP for astrocytes) was performed at DIV35 (Figure 1A). Quantitative analysis revealed that the SC cultures were composed of 44.2 ± 5.2% OPC/oligodendrocytes, 30.2 ± 2.0% neurons, 16.3 ± 4.0% microglia, and 9.2 ± 1.9% astrocytes (Figure 1B). In BC cultures, the composition was 28.6 ± 2.9% OPC/oligodendrocytes, 23.4 ± 3.1% neurons, 9.7 ± 0.9% microglia, and 38.4 ± 4.1% astrocytes, as shown in Figure 1B. Analyses at longer times (DIV45) showed a substantial reduction in the percentage of neurons, both in SC and BC cultures (Figure 1C), with total cell numbers remaining unchanged (data not shown). Furthermore, the quantification of the myelination rate revealed that, at DIV35, the majority of neurons were myelinated in SC (59.4 ± 18.3%) and BC (52.0 ± 9.8%) cultures. At DIV45, a significant decrease in the percentage of myelinated neurons in both SC (26.4 ± 3.9%) and BC (22.0 ± 2.7%) cultures was observed, with an increase in the ratio of unmyelinated to myelinated cells (Figures 1D,E). These results indicate that the optimal time to study demyelination in these cultures is approximately DIV35.

**Figure 1:**
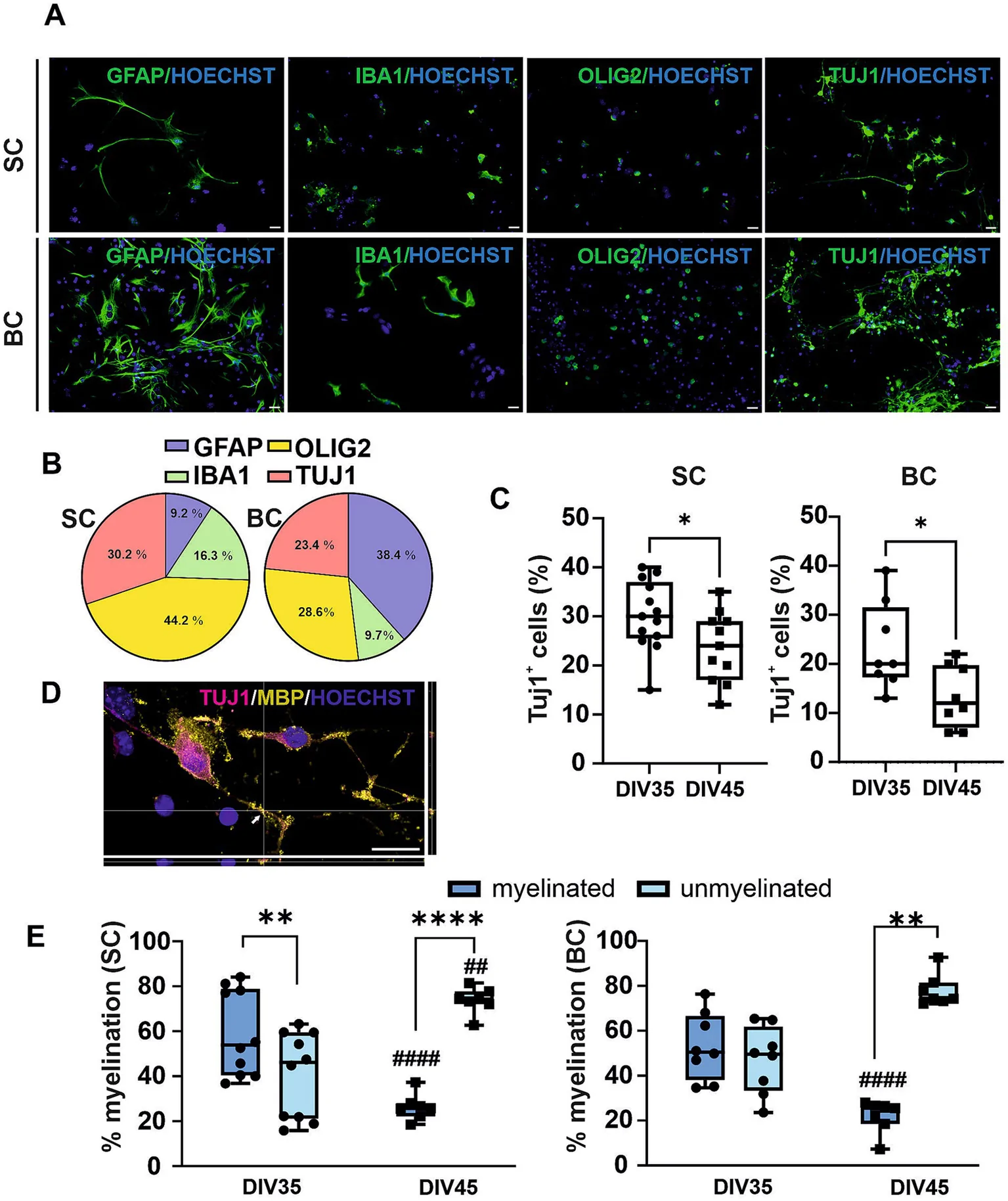
Characterization of spinal cord (SC) and brain cortex (BC) cell cultures from postnatal mice. **(A)** SC and BC cultures were analyzed for cell composition by immunofluorescence. Astrocytes, microglia, oligodendrocytes/OPCs, and neurons were recognized, respectively, by GFAP, Iba1, Olig2, and Tuj1 staining (in green); nuclei were stained with Hoechst (in blue); scale bar = 20 μm. **(B)** Quantification of *n* = 4 independent experiments; data are reported as % of each cell population in relation to the total number of cells. In SC, % of cells positive for GFAP = 9.2 ± 1.9, for Iba1 = 16.3 ± 4.0, for Olig2 = 44.2 ± 5.2, and for Tuj1 = 30.2 ± 2.0; in BC, % of cells positive for GFAP = 38.4 ± 4.1, for Iba1 = 9.7 ± 0.9, for Olig2 = 28.6 ± 2.9, and for Tuj1 = 23.4 ± 3.1. **(C)** SC and BC cell cultures were analyzed for the % of Tuj1^+^ cells in relation to the total number of cells at DIV35 and 45. Data are reported as % mean of Tuj1 + cells ± SE, for SC: Unpaired *t* test, * *p* = 0.0361; for DIV35 *n* = 13, for DIV45 *n* = 11; for BC: Unpaired *t* test, * *p* = 0.0208 for DIV35 *n* = 8, for DIV45 *n* = 8. **(D)** Representative confocal image of primary neuronal culture immunostained for MBP (yellow) and Tuj1 (magenta, Hoechst in blue; scale bar = 10 μm). The image shows a single optical plane (0.25 μm) of the acquired *z*-stack (depth of 3.75 μm), with orthogonal projections along the XZ (above) and YZ (right) axes. The orthogonal projections illustrate the three-dimensional distribution and colocalization of MBP around the Tuj1^+^ neurite (arrow). **(E)** SC and BC cell cultures were analyzed for myelination at DIV35 and 45. Data are reported as % of myelination ± SE; for SC, *n* = 10: Two-way ANOVA followed by Tukey’s multiple comparison test, ** *p* = 0.0024 for myelinated vs. unmyelinated at DIV35, **** *p* < 0.0001 for myelinated vs. unmyelinated at DIV45, #### *p* < 0.0001 for DIV35 vs. 45 in myelinated and # *p* = 0.0359 for DIV35 vs. 45 in unmyelinated; for BC *n* = 7: Two-way ANOVA followed by Tukey’s multiple comparison test, ns *p* = 0.5140 for myelinated vs. unmyelinated at DIV35, ** *p* = 0.0017 for myelinated vs. unmyelinated at DIV45, #### *p* < 0.0001 for DIV35 vs. 45 in myelinated and ns *p* = 0.7756 for DIV35 vs. 45 in unmyelinated.

**Figure 2:**
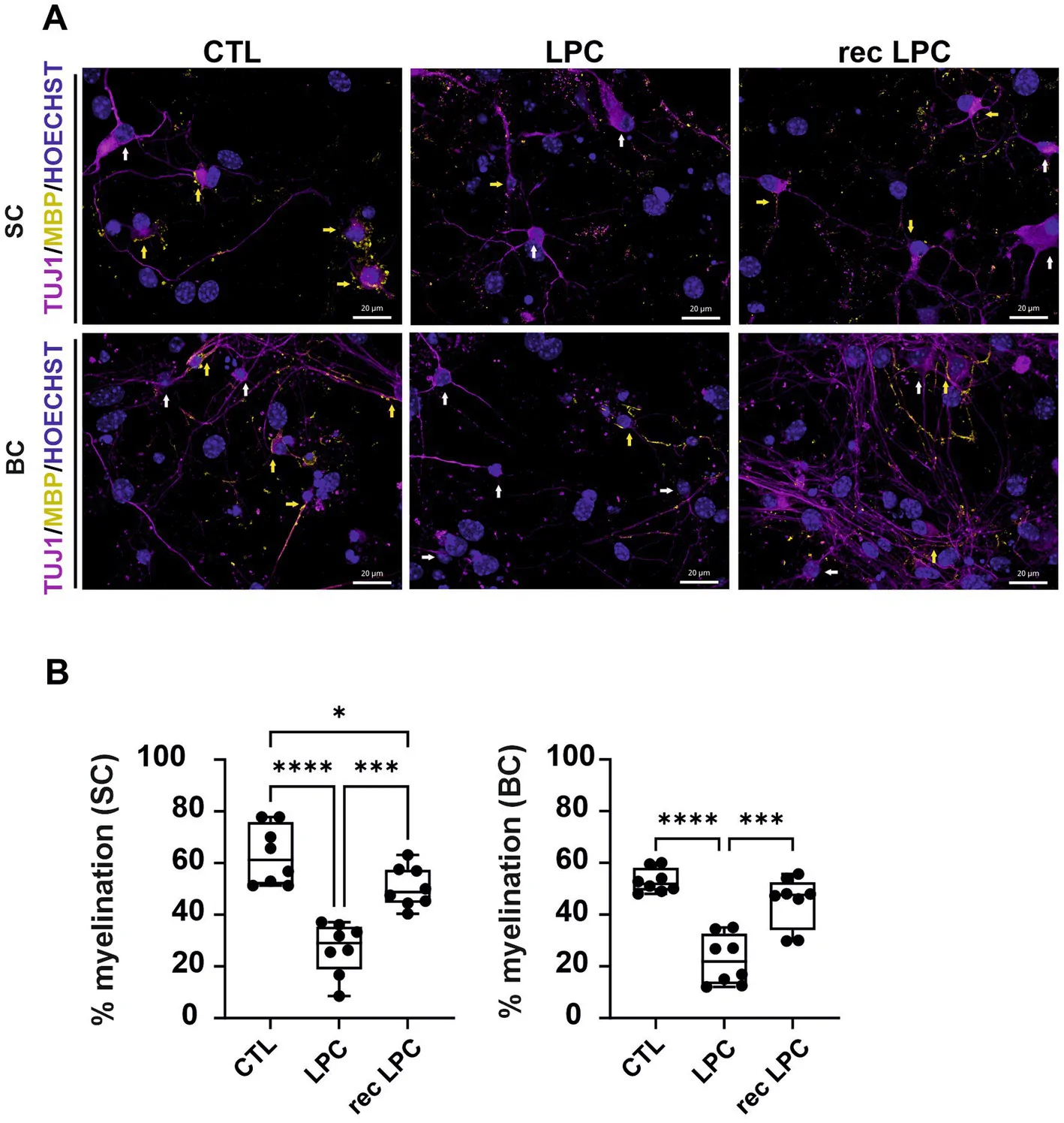
Demyelination/remyelination in spinal cord (SC) and brain cortex (BC) cell cultures from postnatal mice. **(A)** Confocal images of SC and BC cultures were assayed by immunofluorescence for MBP (in yellow) and Tuj1 (in magenta), untreated (CTL), LPC-treated, and recovered upon LPC treatment (rec LPC). Nuclei were stained with Hoechst (in blue); scale bar = 20 μm. White arrows indicate Tuj1^+^/MBP^–^ cells, and yellow arrows indicate Tuj1^+^/MBP^+^ cells. **(B)** Quantification of myelination, data are reported as % myelination mean ± SE, *n* = 8; for SC: One-way ANOVA followed by Tukey’s multiple comparison test, **** *p* < 0.0001 for CTL vs. LPC, * *p* = 0.0295 for CTL vs. rec LPC, *** *p* = 0.0008 for LPC vs. rec LPC; for BC: One-way ANOVA followed by Tukey’s multiple comparison test **** *p* < 0.0001 for CTL vs. LPC, ns *p* = 0.219 for CTL vs. rec LPC, *** *p* = 0.0008 for LPC vs. rec LPC.

To induce demyelination, cultures at DIV29–34 were treated with lysophosphatidylcholine (LPC) for 24 h (Birgbauer et al., 2004). This treatment significantly reduced the percentage of myelinated neurons: in SC cultures from 62.4 ± 4.3% (control) to 29.2 ± 3.8% (LPC) and in BC cultures from 52.6 ± 2.5% (control) to 22.7 ± 4.9% (LPC). Ethanol, used as a vehicle to dissolve LPC, did not affect myelination when incubated alone for 24 h (60.3 ± 11.1 in SC cultures, *p* = 0.657 vs. control; 52.4 ± 6.3 in BC cultures, *p* = 0.819 vs. control; data not shown). After 7 days, a process of spontaneous remyelination takes place, with a complete recovery in BC cultures (to 45.2 ± 5.5%) and a partial recovery in SC cultures (to 49.8 ± 2.1%, *p* = 0.0425), as shown in Figures 2A,B. Given this incomplete recovery in SC cultures, only these preparations were subsequently used to assess the effect of ibrutinib, as detailed in the following paragraph.

### Ibrutinib induced complete recovery of myelination after LPC damage

Bruton’s tyrosine kinase is a non-receptor cytoplasmic protein activated by a plethora of other receptors (i.e., G-protein-coupled receptors, B-cell receptors, interleukin receptors, and tyrosine kinase receptors; Heinonen et al., 2002) and, within the CNS, it is mainly expressed by CD11b^+^ cells (Elkjaer et al., 2023). Its blockade promotes myelin repair (Martin et al., 2020). Among the BTK inhibitors, ibrutinib is a selective and irreversible inhibitor that binds to the kinase domain (Honigberg et al., 2010; de Porto et al., 2019), reducing inflammation and promoting neuroprotection in both *in vitro* and *in vivo* models (Nam et al., 2018; Yu et al., 2021). We investigated ibrutinib-induced remyelination in postnatal SC cultures and found that it enhances recovery in SC cultures where spontaneous remyelination is insufficient, without further increasing myelination in BC cultures (Figure 3A). DMSO, used as a vehicle to dissolve ibrutinib, did not affect myelination. In SC cultures, myelination levels were comparable to those observed after LPC (50.7 ± 2.3% vs. 52.4 ± 2.1%, *p* = 0.591; data not shown), as well as in BC cultures (48.1 ± 2.3% vs. 47.7 ± 2.8%, *p* = 0.919; data not shown). BTK plays a pivotal role in a variety of immune cells, including microglial cells, where it elicits inflammatory responses (Gruber et al., 2024) and reduces the release of Ca^2+^ from the endoplasmic reticulum (Yu et al., 2021; Di Paolo et al., 2011). Since ibrutinib has a direct anti-inflammatory effect on BV2 microglia-like (Chang et al., 2011) cells treated with LPS (Nam et al., 2018), we used these cells to determine whether microglia are involved in the process of neuronal remyelination induced by ibrutinib. We first demonstrated that LPC treatment of these cells does not induce changes in the expression of pro-inflammatory factors, such as *iNOS* and *TNFα*, due to the absence of neurons and oligodendrocytes in the BV2 cultures (Plemel et al., 2018; Figure 3B). To induce an inflammatory phenotype in BV2 cells, they were treated with LPS for 24 h, and the content of the extracellular vesicles (EVs) released in the presence and absence of ibrutinib was analyzed. LPS treatment was used to generate EVs carrying a microglia-derived inflammatory signature (Yang et al., 2018; Grimaldi et al., 2019), enabling us to assess how ibrutinib modulates this response. Data demonstrated that ibrutinib treatment reduced the number of EVs released by these cells, without affecting the size profile (mean size 157.9 ± 1.1 nm vs. 159.6 ± 3.2 nm, *n* = 3, *p* = 0.445; Figure 3C,D). We next evaluated the functional impact of these EVs on myelination recovery in SC cultures. EVs derived from LPS-treated BV2 cells did not improve myelination following LPC-induced demyelination, indicating that inflammatory EVs do not promote repair. In contrast, EVs obtained from BV2 cells treated with LPS and ibrutinib significantly enhanced myelination. This pro-myelinating effect persisted even when endogenous EV release in SC cultures was inhibited with calpeptin, confirming that the beneficial action of EVs derived from ibrutinib-treated microglial cells is mediated by the exogenously applied vesicles (Figure 3E). This result suggested that the pharmacological modulation of microglia alters EV cargo in a manner that could support remyelination. For that reason, we carried out miRNA analyses of EVs isolated from LPS-treated BV2 cells. The analyses revealed the presence of two miRNAs associated with inflammation and remyelination, miR-223 and miR-155 (Mycko and Baranzini, 2020; Galloway et al., 2019). Quantification of miRNAs within EVs showed that ibrutinib treatment increased EV-associated miR-223 levels, while miR-155 remained unchanged (Figure 3F). Taking advantage of the miRCarta database (version 1.1), we identified stathmin-1 (*STMN1*) as a possible target of miR-223 in oligodendrocytes. Stathmin-1 is a microtubule-destabilizing protein that inhibits tubulin polymerization, increases cytoskeletal stiffness, and is overexpressed in oligodendrocytes derived from MS patients (Liu et al., 2005; Belmont and Mitchison, 1996). Interestingly, ibrutinib treatment of primary SC cultures recovering from LPC-induced demyelination decreased *STMN1* levels (Figure 3G), providing preliminary evidence of a putative mechanism underlying ibrutinib-induced remyelination.

**Figure 3:**
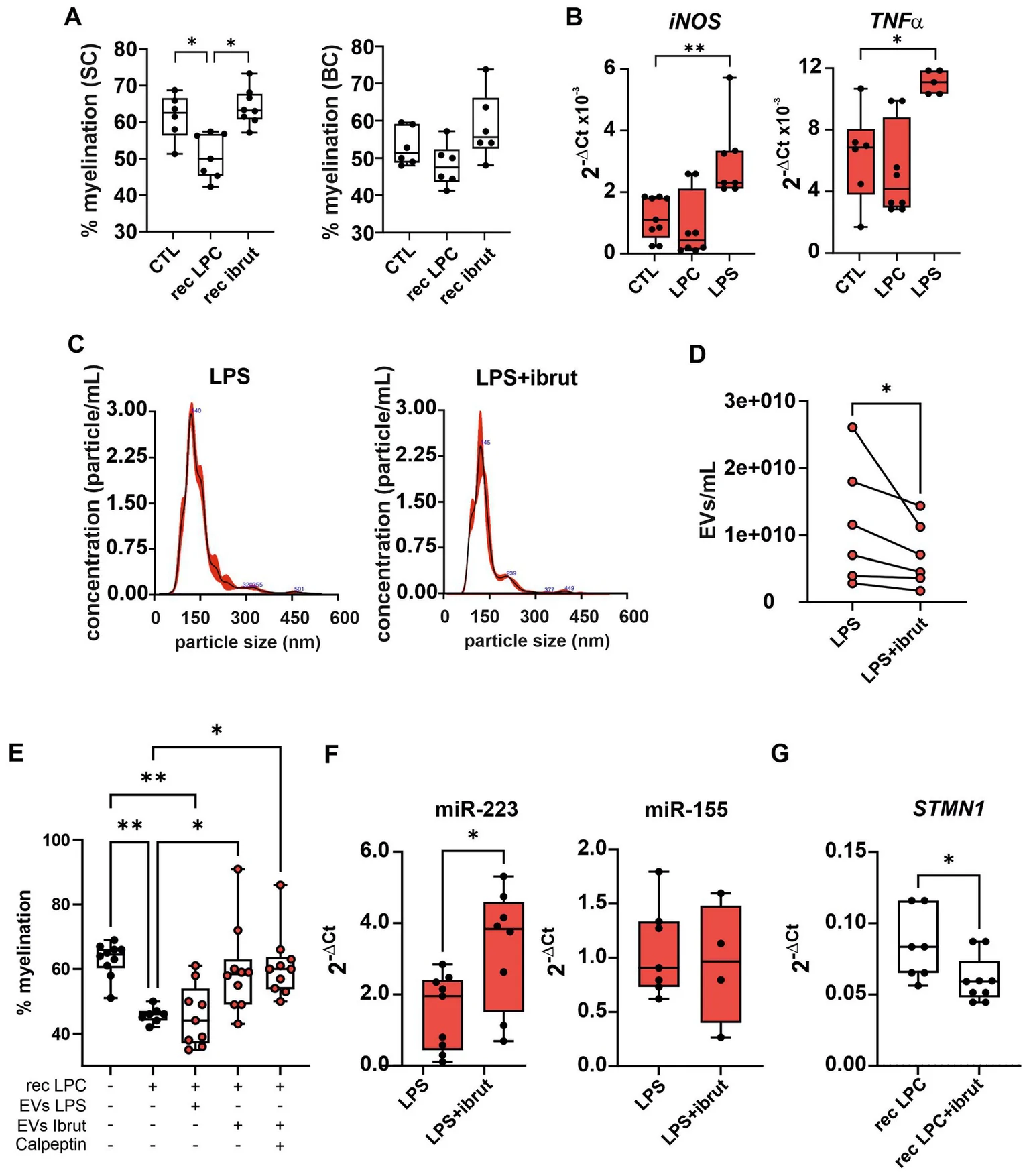
Effects of ibrutinib on myelination after LPC damage. **(A)** Quantification of myelination in controls (CTL), recovered (rec LPC) and recovered plus ibrutinib (rec ibrut) spinal cord (SC) and brain cortex (BC) cultures; data are reported as % myelination, mean ± SE; for SC (CTL, *n* = 6; rec LPC, *n* = 7; rec ibrut, *n* = 8): One-way ANOVA followed by Tukey’s multiple comparison test, * *p* = 0.05 for CTL vs. rec LPC, ns *p* = 0.9528 for CTL vs. rec ibrut, * *p* = 0.0415 for rec LPC vs. rec ibrut; for BC (*n* = 6): One-way ANOVA followed by Tukey’s multiple comparison test, ns *p* = 0.5573 for CTL vs. LPC, ns *p* = 0.5048 for CTL vs. rec ibrut, ns *p* = 0.1164 for rec LPC vs. ibrut. **(B)** Quantitative real-time PCR of *iNOS* and *TNFα* gene expression in CTL, LPC, and LPS-treated BV2 microglial-like cells. Each sample was normalized to *GAPDH* mRNA. Data were expressed as the mean ± SEM of 2^–∆Ct^, *n* = 5–9. Data were analyzed by one-way ANOVA followed by Tukey’s multiple comparison test, ** *p* = 0.0032 for CTL vs. LPS; ns *p* = 0.8057 for CTL vs. LPC for *iNOS*; * *p* = 0.0149 for CTL vs. LPS; ns *p* = 0.7228 for CTL vs. LPC for *TNFα*. **(C)** Representative concentration/size graph from the nanoparticle tracking analysis (NTA) on EVs derived from BV2 cells treated with LPS or LPS plus ibrutinib (LPS + ibrut), illustrative of *n* = 3 independent experiments. **(D)** Quantification of EVs derived from BV2 cells treated with LPS or LPS plus ibrutinib (LPS + ibrut). Data are reported as particle number mean ± SEM, *n* = 6 independent experiments, Wilcoxon matched-pairs rank test, * *p* = 0.0312. **(E)** Quantification of myelination in control (first histogram) and recovered (rec LPC) spinal cord (SC) cultures following treatment with EVs derived from BV2 cells. EVs were obtained from BV2 stimulated with LPS (EVs LPS) or with LPS plus ibrutinib (EVs ibrut). Calpeptin was applied to SC cultures prior to LPC exposure to inhibit endogenous EV release (calpeptin). Data are reported as % myelination (mean ± SE), *n* = 3 independent experiments; analyses were performed using one-way ANOVA followed by Tukey’s multiple comparison test. Significant pairwise comparisons were as follows: CTL vs. rec LPC, **p = 0.0047; CTL vs. rec LPC + EVs LPS, **p = 0.0019; rec LPC vs. rec LPC + EVs ibrut, *p = 0.0358; rec LPC vs. rec LPC + calpeptin + EVs ibrut, *p = 0.0156; rec LPC + EVs LPS vs. rec LPC + EVs ibrut, *p* = 0.0188; rec LPC + EVs LPS vs. rec LPC + calpeptin + EVs ibrut, *p* = 0.0073. **(F)** Quantitative real-time PCR of miR-223 and miR-155 expression in LPS and LPS plus ibrutinib (LPS + ibrut)-treated BV2 microglial-like cells. Each sample was normalized to U6 snRNA. Data were expressed as the mean ± SEM of 2^–∆Ct^, *n* = 4–9, unpaired *t*-test, * *p* = 0.0172 for miR-223; ns *p* = 0.6992 for miR-155. **(G)** Quantitative real-time PCR of *STMN1* gene expression in SC cultures recovering from LPC treatment (rec LPC) and in SC cultures recovering from LPC treatment and subsequently exposed to ibrutinib (rec LPC + ibrut). Each sample was normalized to *GAPDH* mRNA. Data were expressed as the mean ± SEM of 2^–∆Ct^; LPC, *n* = 7 vs. LPC + ibrut, *n* = 9, unpaired *t*-test, * *p* = 0.0385.

## Discussion

In this study, we used postnatal mouse cell cultures from the spinal cord and the brain cortex to investigate myelination and remyelination upon LPC and BTK inhibitor treatment. We demonstrated that LPC induces demyelination of both SC and BC neurons and confirmed that ibrutinib accelerates remyelination, possibly with the contribution of microglial cells. As a consequence of demyelination, microglial cells acquire an inflamed phenotype (Marzan et al., 2021). We mimicked microglial inflammation by treating BV2 cells with LPS and observed that ibrutinib reduces both the total number of EVs released by these cells and alters their content, with an increase in miR-223 expression. miR-223 has been shown to regulate the expression of stmn1 mRNA, which encodes the microtubule-destabilizing protein Stathmin-1 (Birnie et al., 2015), previously reported to be overexpressed in oligodendrocytes of MS patients (Belmont and Mitchison, 1996), and we observed that LPC-ibrutinib-treated SC cells significantly reduced *STMN1* expression.

To obtain postnatal mouse neuronal cultures from the SC and BC and to investigate the remyelination process, we modified the protocol described by Pang et al. (2012) using P0-P2 mice instead of rat embryos. This modification allows us to better align with the 3Rs principles as advocated by the EU Directive 26/2010. The protocol was applied to tissues derived from both the SC and BC, revealing no significant differences in the non-neuron/neuron cell ratio (~3:1). This ratio is consistent with the cellular composition of these regions in mice and other species (i.e., monkeys and humans; Bjugn, 1993; Bjugn and Gundersen, 1993). In contrast to embryo-derived cultures, where the percentage of myelination remained constant beyond 40 DIV (Pang et al., 2012; Fu et al., 2013), in postnatal cell cultures, we observed a significant reduction in myelination at DIV45 and a lower ratio of myelinated/unmyelinated cells. For these reasons, we decided to use cultures that were not older than DIV35.

To induce demyelination, we used the LPC, which causes disruption of the myelin sheath (Ou et al., 2024; Plemel et al., 2017), with a primary myelinopathy induced by damage to the oligodendrocyte lipid bilayer and a secondary immune response (Plemel et al., 2018). LPC-induced demyelination mimics the pathological features of different myelinopathies such as MS, and it is spontaneously reversible (Plemel et al., 2018; Law et al., 2019), allowing us to study both demyelination and subsequent remyelination processes in controlled settings. We confirmed that LPC can induce a reversible demyelination in postnatal SC and BC cultures and that ibrutinib treatment accelerated remyelination. BTKi are currently under investigation for their use in MS patients due to their capacity to contrast the MS disease course (Himmelbauer et al., 2024; Montalban et al., 2019; Nuesslein-Hildesheim et al., 2023; Reich et al., 2021; Rozkiewicz et al., 2023). These drugs directly target B cells and microglia, and their effects in MS patients could be due to the downregulation of proinflammatory markers (Greenberg, 2024; Nam et al., 2018). While some studies have suggested that pro-inflammatory signals can initiate the repair process, thereby activating myeloid cells to clear myelin debris and to release factors that stimulate oligodendrocyte precursor cells (OPCs; Cunha et al., 2020), chronic inflammation inhibits OPC differentiation and remyelination (Höftberger and Lassmann, 2018; Zveik et al., 2024). The impact of an inflamed environment on remyelination remains a subject of ongoing debate (Voet et al., 2019).

We and others have demonstrated that EV cargo recapitulates the inflammatory state of the donor cells, contributing to the transfer of proinflammatory and anti-inflammatory phenotypes (Grimaldi et al., 2019; Ortega-Pineda et al., 2022; Rinaldi et al., 2024). It has been shown that microglial EVs modulate the response of both OPCs and astrocytes in the case of myelin lesions (Lombardi et al., 2019).

We demonstrated that ibrutinib reduced the number of BV2-derived EVs upon LPS treatment. A similar modulation has been previously reported in different cell types, likely suggesting a blockade of pathways involved in vesicle release (Hsu et al., 2013; Rutman et al., 2018).

Notably, the effect of ibrutinib on myelination recovery is attributable to its capacity to modulate EV release from microglia; indeed, administering ibrutinib to BV2 cells and treating SC cultures with EVs derived from these microglia-like cells resulted in recovery comparable to that observed when ibrutinib was applied directly to spinal cord cultures. Importantly, this recovery occurred even when endogenous vesicle release is blocked by calpeptin, indicating that the effect is independent of modulation of endogenous EV release.

Given that this effect cannot be attributed to the quantity of EVs, we hypothesized that their content was modified. Therefore, we investigated the presence in EVs of miRNAs potentially involved in remyelination: miRNA-155 and miR-223. miR-155 is a proinflammatory miRNA, being relevant in myeloid cell polarization in MS (Hu et al., 2022; Moore et al., 2013) and in LPS-treated BV2 cells; miR-155 increases the release of the pro-inflammatory cytokine IL1β (Yin et al., 2017). On the other hand, miR-223, which is upregulated in MS patients compared to healthy individuals (Hosseini et al., 2016), exerts an anti-inflammatory role, inducing the activation of macrophages, neutrophils, and other immune cells (Zhuang et al., 2012; Gou et al., 2018; Chen et al., 2008; Dorhoi et al., 2013; Satoorian et al., 2016). In the experimental model of MS, experimental autoimmune encephalomyelitis (EAE), miR-223 has neuroprotective roles, and miRNA-223 knockout mice developed less severe EAE (Morquette et al., 2019; Cantoni et al., 2017). In microglia, miRNA-223 accelerates debris clearance after lysolecithin-induced demyelination (Ou et al., 2024). Our results show that EVs released by LPS/ibrutinib-treated BV2 cells expressed the same amount of miR-155, while miR-223 was upregulated. Interestingly, one of the targets of miR-223 is *STMN1*, a gene coding for stathmin-1, a protein overexpressed in MS patients (Liu et al., 2005) and involved in tubulin polymerization (Belmont and Mitchison, 1996). Of note, tubulin has diagnostic value in MS (Madeddu et al., 2013).

To summarize, our study adds postnatal SC cultures as a tool to investigate demyelination *in vitro* and provides preliminary evidence of a potential role of ibrutinib in reducing the microglial-mediated inflammatory microenvironment upon demyelination.

## Data Availability

The raw data supporting the conclusions of this article will be made available by the authors, without undue reservation.

## References

[ref1] Baumann N. Pham-Dinh D. (2001). Biology of oligodendrocyte and myelin in the mammalian central nervous system. Physiol. Rev. 81, 871–927. doi: 10.1152/physrev.2001.81.2.871, 11274346

[ref2] Belmont L. D. Mitchison T. J. (1996). Identification of a protein that interacts with tubulin dimers and increases the catastrophe rate of microtubules. Cell 84, 623–631. 10.1016/s0092-8674(00)81037-5 8598048

[ref3] Birgbauer E. Rao T. S. Webb M. (2004). Lysolecithin induces demyelination in vitro in a cerebellar slice culture system. J. Neurosci. Res. 78, 157–166. doi: 10.1002/jnr.20248, 15378614

[ref4] Birnie K. A. Yip Y. Y. Ng D. C. Kirschner M. B. Reid G. Prêle C. M. . (2015). Loss of miR-223 and JNK signaling contribute to elevated Stathmin in malignant pleural mesothelioma. Mol. Cancer Res. 13, 1106–1118. doi: 10.1158/1541-7786.MCR-14-0442, 25824152

[ref5] Bjugn R. (1993). The use of the optical disector to estimate the number of neurons, glial and endothelial cells in the spinal cord of the mouse--with a comparative note on the rat spinal cord. Brain Res. 627, 25–33. 10.1016/0006-8993(93)90744-8 8293301

[ref6] Bjugn R. Gundersen H. J. (1993). Estimate of the total number of neurons and glial and endothelial cells in the rat spinal cord by means of the optical disector. J. Comp. Neurol. 328, 406–414. 10.1002/cne.903280307 8440788

[ref7] Branchi I. Alboni S. Maggi L. (2014). The role of microglia in mediating the effect of the environment in brain plasticity and behavior. Front. Cell. Neurosci. 8:8. doi: 10.3389/fncel.2014.00390, 25477783 PMC4235360

[ref8] Brunner C. Müller B. Wirth T. (2005). Bruton’s tyrosine kinase is involved in innate and adaptive immunity. Histol. Histopathol. 20, 945–955. doi: 10.14670/HH-20.945, 15944945

[ref9] Cantoni C. Cignarella F. Ghezzi L. Mikesell B. Bollman B. Berrien-Elliott M. M. . (2017). Mir-223 regulates the number and function of myeloid-derived suppressor cells in multiple sclerosis and experimental autoimmune encephalomyelitis. Acta Neuropathol (Berl). 133, 61–77. doi: 10.1007/s00401-016-1621-6, 27704281 PMC5423756

[ref10] Catalano M. O'Driscoll L. (2019). Inhibiting extracellular vesicles formation and release: a review of EV inhibitors. J. Extracell. Vesicles 9:1703244. doi: 10.1080/20013078.2019.1703244 PMC6968539 32002167

[ref11] Chang B. Y. Huang M. M. Francesco M. Chen J. Sokolove J. Magadala P. . (2011). The Bruton tyrosine kinase inhibitor PCI-32765 ameliorates autoimmune arthritis by inhibition of multiple effector cells. Arthritis Res. Ther. 13:R115. doi: 10.1186/ar3400, 21752263 PMC3239353

[ref12] Chen R. Cros D. Curra A. Di Lazzaro V. Lefaucheur J.-P. Magistris M. R. . (2008). The clinical diagnostic utility of transcranial magnetic stimulation: report of an IFCN committee. Clin. Neurophysiol. 119, 504–532. doi: 10.1016/j.clinph.2007.10.014, 18063409

[ref13] Cherry J. D. Olschowka J. A. O’Banion M. K. (2014). Neuroinflammation and M2 microglia: the good, the bad, and the inflamed. J. Neuroinflammation 11:98. doi: 10.1186/1742-2094-11-98, 24889886 PMC4060849

[ref14] Cunha M. I. Su M. Cantuti-Castelvetri L. Müller S. A. Schifferer M. Djannatian M. . (2020). Pro-inflammatory activation following demyelination is required for myelin clearance and oligodendrogenesis. J. Exp. Med. 217:e20191390. doi: 10.1084/jem.20191390, 32078678 PMC7201919

[ref15] de Porto A. P. Liu Z. de Beer R. Florquin S. de Boer O. J. Hendriks R. W. . (2019). Btk inhibitor ibrutinib reduces inflammatory myeloid cell responses in the lung during murine pneumococcal pneumonia. Mol Med Camb Mass. 25:3. doi: 10.1186/s10020-018-0069-7, 30646846 PMC6332549

[ref16] Di Paolo J. A. Huang T. Balazs M. (2011). Specific Btk inhibition suppresses B cell- and myeloid cell-mediated arthritis. Nat. Chem. Biol. 7, 41–50. doi: 10.1038/nchembio.481, 21113169

[ref17] Dorhoi A. Iannaccone M. Farinacci M. Faé K. C. Schreiber J. Moura-Alves P. . (2013). MicroRNA-223 controls susceptibility to tuberculosis by regulating lung neutrophil recruitment. J. Clin. Invest. 123, 4836–4848. doi: 10.1172/JCI67604, 24084739 PMC3809781

[ref18] Dybowski S. Torke S. Weber M. S. (2023). Targeting B cells and microglia in multiple sclerosis with Bruton tyrosine kinase inhibitors: a review. JAMA Neurol. 80, 404–414. doi: 10.1001/jamaneurol.2022.5332, 36780171

[ref19] Eldeiry M. Yamanaka K. Reece T. B. Aftab M. (2017). Spinal cord neurons isolation and culture from neonatal mice. J. Vis. Exp. 125:55856. doi: 10.3791/55856 PMC5612298 28745627

[ref20] Elkjaer M. L. Waede M. R. Kingo C. Damsbo K. Illes Z. (2023). Expression of Bruton’s tyrosine kinase in different type of brain lesions of multiple sclerosis patients and during experimental demyelination. Front. Immunol. 14:1264128. doi: 10.3389/fimmu.2023.1264128, 38022591 PMC10679451

[ref21] Fox J. E. Austin C. D. Reynolds C. C. Steffen P. K. (1991). Evidence that agonist-induced activation of calpain causes the shedding of procoagulant-containing microvesicles from the membrane of aggregating platelets. J. Biol. Chem. 266, 13289–13295. 2071604

[ref22] Franklin R. J. M. ffrench-Constant C. (2008). Remyelination in the CNS: from biology to therapy. Nat. Rev. Neurosci. 9, 839–855. doi: 10.1038/nrn2480, 18931697

[ref23] Fu Y. Rusznák Z. Herculano-Houzel S. Watson C. Paxinos G. (2013). Cellular composition characterizing postnatal development and maturation of the mouse brain and spinal cord. Brain Struct. Funct. 218, 1337–1354. doi: 10.1007/s00429-012-0462-x, 23052551

[ref24] Galloway D. A. Blandford S. N. Berry T. Williams J. B. Stefanelli M. Ploughman M. . (2019). miR-223 promotes regenerative myeloid cell phenotype and function in the demyelinated central nervous system. Glia 67, 857–869. doi: 10.1002/glia.23576, 30548333

[ref25] Gou W. Zhang Z. Yang C. Li Y. (2018). MiR-223/Pknox1 axis protects mice from CVB3-induced viral myocarditis by modulating macrophage polarization. Exp. Cell Res. 366, 41–48. doi: 10.1016/j.yexcr.2018.03.004, 29524390

[ref26] Greenberg B. M. (2024). Bruton’s tyrosine kinase inhibitors for multiple sclerosis treatment. Neurol. Clin. 42, 155–163. doi: 10.1016/j.ncl.2023.07.006, 37980113

[ref27] Grimaldi A. Serpe C. Chece G. Nigro V. Sarra A. Ruzicka B. . (2019). Microglia-derived microvesicles affect microglia phenotype in glioma. Front. Cell. Neurosci. 13:41. doi: 10.3389/fncel.2019.00041, 30853898 PMC6395438

[ref28] Gruber R. C. Wirak G. S. Blazier A. S. Lee L. Dufault M. R. Hagan N. . (2024). BTK regulates microglial function and neuroinflammation in human stem cell models and mouse models of multiple sclerosis. Nat. Commun. 15:10116. doi: 10.1038/s41467-024-54430-8, 39578444 PMC11584639

[ref29] Ha M. Kim V. N. (2014). Regulation of microRNA biogenesis. Nat. Rev. Mol. Cell Biol. 15, 509–524. doi: 10.1038/nrm3838, 25027649

[ref30] Heinonen J. E. Smith C. I. E. Nore B. F. (2002). Silencing of Bruton’s tyrosine kinase (Btk) using short interfering RNA duplexes (siRNA). FEBS Lett. 527, 274–278. doi: 10.1016/s0014-5793(02)03206-4, 12220673

[ref31] Himmelbauer M. K. Bajrami B. Basile R. Capacci A. Chen T. Y. Choi C. K. . (2024). Discovery and preclinical characterization of BIIB129, a covalent, selective, and brain-penetrant BTK inhibitor for the treatment of multiple sclerosis. J. Med. Chem. 67, 8122–8140. doi: 10.1021/acs.jmedchem.4c00220, 38712838 PMC11129193

[ref32] Höftberger R. Lassmann H. (2018). Inflammatory demyelinating diseases of the central nervous system. Handb. Clin. Neurol. 145, 263–283. doi: 10.1016/B978-0-12-802395-2.00019-5, 28987175 PMC7149979

[ref33] Honigberg L. A. Smith A. M. Sirisawad M. Verner E. Loury D. Chang B. . (2010). The Bruton tyrosine kinase inhibitor PCI-32765 blocks B-cell activation and is efficacious in models of autoimmune disease and B-cell malignancy. Proc. Natl. Acad. Sci. USA 107, 13075–13080. doi: 10.1073/pnas.1004594107, 20615965 PMC2919935

[ref34] Hosseini A. Ghaedi K. Tanhaei S. GanjalikhaniHakemi M. Teimuri S. Etemadifar M. (2016). Upregulation of CD4T-cell derived miR-223 in the relapsing phase of multiple sclerosis patients. Cell J. (Yakhteh) 18:4565. doi: 10.22074/cellj.2016.4565 PMC5011325 27602319

[ref35] Hsu J. Gu Y. Tan S. L. Narula S. DeMartino J. A. Liao C. (2013). Bruton’s tyrosine kinase mediates platelet receptor-induced generation of microparticles: a potential mechanism for amplification of inflammatory responses in rheumatoid arthritis synovial joints. Immunol. Lett. 150, 97–104. doi: 10.1016/j.imlet.2012.12.007, 23266841

[ref36] Hu J. Huang S. Liu X. Zhang Y. Wei S. Hu X. (2022). miR-155: an important role in inflammation response. J. Immunol. Res. 2022, 1–13. doi: 10.1155/2022/7437281, 35434143 PMC9007653

[ref37] Jastorff A. M. Haegler K. Maccarrone G. Holsboer F. Weber F. Ziemssen T. . (2009). Regulation of proteins mediating neurodegeneration in experimental autoimmune encephalomyelitis and multiple sclerosis. Proteomics Clin. Appl. 3, 1273–1287. doi: 10.1002/prca.200800155, 21136950

[ref38] Law S. H. Chan M. L. Marathe G. K. Parveen F. Chen C. H. Ke L. Y. (2019). An updated review of Lysophosphatidylcholine metabolism in human diseases. Int. J. Mol. Sci. 20:1149. doi: 10.3390/ijms20051149, 30845751 PMC6429061

[ref39] Liu A. Stadelmann C. Moscarello M. Bruck W. Mastronardi F. G. Casaccia-Bonnefil P. (2005). Expression of stathmin, a developmentally controlled cytoskeleton-regulating molecule, in demyelinating disorders. J. Neurosci. 25, 737–747. doi: 10.1523/JNEUROSCI.4174-04.2005, 15659612 PMC6725324

[ref40] Lombardi M. Parolisi R. Scaroni F. Bonfanti E. Gualerzi A. Gabrielli M. . (2019). Detrimental and protective action of microglial extracellular vesicles on myelin lesions: astrocyte involvement in remyelination failure. Acta Neuropathol (Berl). 138, 987–1012. doi: 10.1007/s00401-019-02049-1, 31363836 PMC6851224

[ref41] Love S. (2006). Demyelinating diseases. J. Clin. Pathol. 59, 1151–1159. doi: 10.1136/jcp.2005.031195, 17071802 PMC1860500

[ref42] Madeddu R. Farace C. Tolu P. Solinas G. Asara Y. Sotgiu M. A. . (2013). Cytoskeletal proteins in the cerebrospinal fluid as biomarker of multiple sclerosis. Neurol. Sci. 34, 181–186. doi: 10.1007/s10072-012-0974-4, 22362332

[ref43] Martin E. Aigrot M. S. Grenningloh R. Stankoff B. Lubetzki C. Boschert U. . (2020). Bruton’s tyrosine kinase inhibition promotes myelin repair. Brain Plast. 5, 123–133. doi: 10.3233/BPL-200100, 33282676 PMC7685672

[ref44] Marzan D. E. Brügger-Verdon V. West B. L. Liddelow S. Samanta J. Salzer J. L. (2021). Activated microglia drive demyelination via CSF1R signaling. Glia 69, 1583–1604. doi: 10.1002/glia.23980, 33620118 PMC9250806

[ref45] Montalban X. Arnold D. L. Weber M. S. Staikov I. Piasecka-Stryczynska K. Willmer J. . (2019). Placebo-controlled trial of an Oral BTK inhibitor in multiple sclerosis. N. Engl. J. Med. 380, 2406–2417. doi: 10.1056/NEJMoa1901981, 31075187

[ref46] Moore C. S. Rao V. T. S. Durafourt B. A. Bedell B. J. Ludwin S. K. Bar-Or A. . (2013). miR-155 as a multiple sclerosis-relevant regulator of myeloid cell polarization. Ann. Neurol. 74, 709–720. doi: 10.1002/ana.23967, 23818336

[ref47] Morquette B. Juźwik C. A. Drake S. S. Charabati M. Zhang Y. Lécuyer M. A. . (2019). MicroRNA-223 protects neurons from degeneration in experimental autoimmune encephalomyelitis. Brain J. Neurol. 142, 2979–2995. doi: 10.1093/brain/awz245, 31412103

[ref48] Mycko M. P. Baranzini S. E. (2020). microRNA and exosome profiling in multiple sclerosis. Mult. Scler. J. 26, 599–604. doi: 10.1177/1352458519879303, 31965891 PMC7160025

[ref49] Nam H. Y. Nam J. H. Yoon G. Lee J. Y. Nam Y. Kang H. J. . (2018). Ibrutinib suppresses LPS-induced neuroinflammatory responses in BV2 microglial cells and wild-type mice. J. Neuroinflammation 15:271. doi: 10.1186/s12974-018-1308-0, 30231870 PMC6145206

[ref50] Nuesslein-Hildesheim B. Ferrero E. Schmid C. Huck C. Smith P. Tisserand S. . (2023). Remibrutinib (LOU064) inhibits neuroinflammation driven by B cells and myeloid cells in preclinical models of multiple sclerosis. J. Neuroinflammation 20:194. doi: 10.1186/s12974-023-02877-9, 37633912 PMC10463946

[ref51] Ortega-Pineda L. Sunyecz A. Salazar-Puerta A. I. Rincon-Benavides M. A. Alzate-Correa D. Anaparthi A. L. . (2022). Designer extracellular vesicles modulate pro-neuronal cell responses and improve intracranial retention. Adv. Healthc. Mater. 11:e2100805. doi: 10.1002/adhm.202100805, 35014204 PMC9466406

[ref52] Ou Z. Cheng Y. Ma H. Chen K. Lin Q. Chen J. . (2024). miR-223 accelerates lipid droplets clearance in microglia following spinal cord injury by upregulating ABCA1. J. Transl. Med. 22:659. doi: 10.1186/s12967-024-05480-5, 39010173 PMC11247820

[ref53] Pang Y. Zheng B. Kimberly S. L. Cai Z. Rhodes P. G. Lin R. C. S. (2012). Neuron-oligodendrocyte myelination co-culture derived from embryonic rat spinal cord and cerebral cortex. Brain Behav. 2, 53–67. doi: 10.1002/brb3.33, 22574274 PMC3343299

[ref54] Plemel J. R. Liu W. Q. Yong V. W. (2017). Remyelination therapies: a new direction and challenge in multiple sclerosis. Nat. Rev. Drug Discov. 16, 617–634. doi: 10.1038/nrd.2017.115, 28685761

[ref55] Plemel J. R. Michaels N. J. Weishaupt N. Caprariello A. V. Keough M. B. Rogers J. A. . (2018). Mechanisms of lysophosphatidylcholine-induced demyelination: a primary lipid disrupting myelinopathy. Glia 66, 327–347. doi: 10.1002/glia.23245, 29068088

[ref56] Reich D. S. Arnold D. L. Vermersch P. Bar-Or A. Fox R. J. Matta A. . (2021). Safety and efficacy of tolebrutinib, an oral brain-penetrant BTK inhibitor, in relapsing multiple sclerosis: a phase 2b, randomised, double-blind, placebo-controlled trial. Lancet Neurol. 20, 729–738. doi: 10.1016/S1474-4422(21)00237-4, 34418400 PMC8434816

[ref57] Rinaldi A. Balietti M. Principi E. de Luca M. de Felice E. Narcisi F. M. . (2024). BV2-derived extracellular vesicles modulate microglia inflammatory profile, neuronal plasticity, and behavioural performances in late adult mice. Brain Behav. Immun. 122, 58–74. doi: 10.1016/j.bbi.2024.08.012, 39128568

[ref58] Rozkiewicz D. Hermanowicz J. M. Kwiatkowska I. Krupa A. Pawlak D. (2023). Bruton’s tyrosine kinase inhibitors (BTKIs): review of preclinical studies and evaluation of clinical trials. Molecules 28:2400. doi: 10.3390/molecules28052400, 36903645 PMC10005125

[ref59] Rutman A. K. Negi S. Gasparrini M. Hasilo C. P. Tchervenkov J. Paraskevas S. (2018). Immune response to extracellular vesicles from human islets of Langerhans in patients with type 1 diabetes. Endocrinology 159, 3834–3847. doi: 10.1210/en.2018-00649, 30307543

[ref60] Satoorian T. Li B. Tang X. Xiao J. Xing W. Shi W. . (2016). Micro RNA 223 promotes pathogenic T-cell development and autoimmune inflammation in central nervous system in mice. Immunology 148, 326–338. doi: 10.1111/imm.12611, 27083389 PMC4948040

[ref61] Sinyuk M. Williams J. L. (2020). Dissection and isolation of murine glia from multiple central nervous system regions. J. Vis. Exp. 160:1345. doi: 10.3791/61345 PMC10249511 32568252

[ref62] Smith A. B. Smirniotopoulos J. G. (2010). Imaging evaluation of demyelinating processes of the central nervous system. Postgrad. Med. J. 86, 218–229. doi: 10.1136/pgmj.2009.087452, 20354045

[ref63] Vereyken E. J. F. Fluitsma D. M. Bolijn M. J. Dijkstra C. D. Teunissen C. E. (2009). An in vitro model for de- and remyelination using lysophosphatidyl choline in rodent whole brain spheroid cultures. Glia 57, 1326–1340. doi: 10.1002/glia.20852, 19191324

[ref64] Voet S. Prinz M. Van Loo G. (2019). Microglia in central nervous system inflammation and multiple sclerosis pathology. Trends Mol. Med. 25, 112–123. doi: 10.1016/j.molmed.2018.11.005, 30578090

[ref65] Wen Q. Wang Y. Pan Q. Tian R. Zhang D. Qin G. . (2021). MicroRNA-155-5p promotes neuroinflammation and central sensitization via inhibiting SIRT1 in a nitroglycerin-induced chronic migraine mouse model. J. Neuroinflammation 18:287. doi: 10.1186/s12974-021-02342-5, 34893074 PMC8665643

[ref66] Xu W. D. Feng S. Y. Huang A. F. (2022). Role of miR-155 in inflammatory autoimmune diseases: a comprehensive review. Inflamm. Res. 71, 1501–1517. doi: 10.1007/s00011-022-01643-6, 36308539

[ref67] Yang Y. Boza-Serrano A. Dunning C. J. R. Clausen B. H. Lambertsen K. L. Deierborg T. (2018). Inflammation leads to distinct populations of extracellular vesicles from microglia. J. Neuroinflammation 15:168. doi: 10.1186/s12974-018-1204-7 PMC5972400 29807527

[ref68] Yang E. J. Yoon J. H. Chung K. C. (2004). Bruton’s tyrosine kinase phosphorylates cAMP-responsive element-binding protein at serine 133 during neuronal differentiation in immortalized hippocampal progenitor cells. J. Biol. Chem. 279, 1827–1837. doi: 10.1074/jbc.M308722200, 14597636

[ref69] Yin H. Song S. Pan X. (2017). Knockdown of miR-155 protects microglia against LPS-induced inflammatory injury via targeting RACK1: a novel research for intracranial infection. J. Inflamm. 14:17. doi: 10.1186/s12950-017-0162-7, 28804270 PMC5549339

[ref70] Yu C. G. Bondada V. Iqbal H. Moore K. L. Gensel J. C. Bondada S. . (2021). Inhibition of Bruton tyrosine kinase reduces neuroimmune cascade and promotes recovery after spinal cord injury. Int. J. Mol. Sci. 23:355. doi: 10.3390/ijms23010355, 35008785 PMC8745213

[ref71] Zhuang G. Meng C. Guo X. Cheruku P. S. Shi L. Xu H. . (2012). A novel regulator of macrophage activation: miR-223 in obesity-associated adipose tissue inflammation. Circulation 125, 2892–2903. doi: 10.1161/CIRCULATIONAHA.111.087817, 22580331

[ref72] Zveik O. Rechtman A. Ganz T. Vaknin-Dembinsky A. (2024). The interplay of inflammation and remyelination: rethinking MS treatment with a focus on oligodendrocyte progenitor cells. Mol. Neurodegener. 19:53. doi: 10.1186/s13024-024-00742-8 PMC11245841 38997755

